# One-dimensional modeling of heterogeneous catalytic chemical looping steam methane reforming in an adiabatic packed bed reactor

**DOI:** 10.3389/fchem.2023.1295455

**Published:** 2023-11-20

**Authors:** Haris Qayyum, Izzat Iqbal Cheema, Mohsin Abdullah, Muhammad Amin, Imtiaz Afzal Khan, Eui-Jong Lee, Kang Hoon Lee

**Affiliations:** ^1^ Department of Chemical Engineering, University of Engineering and Technology, Lahore, Lahore, Punjab, Pakistan; ^2^ Department of Energy Systems Engineering, NFC Institute of Engineering and Technology, Multan, Pakistan; ^3^ Interdisciplinary Research Center for Hydrogen and Energy Storage (Tire II)-Research and Innovation, King Fahd University of Petroleum and Minerals (KFUPM), Dhahran, Saudi Arabia; ^4^ Department of Civil and Environmental Engineering, Hanyang University, Seoul, Republic of Korea; ^5^ Department of Environmental Engineering, Daegu University, Gyeongsan, Republic of Korea; ^6^ Department of Energy and Environmental Engineering,The Catholic University of Korea, Bucheon-si, Republic of Korea

**Keywords:** hydrogen, chemical looping, reforming, energy, gPROMS, methane reforming

## Abstract

Hydrogen production via chemical looping steam methane reforming (CL-SMR) is among the most promising current technologies. This work presents the development in gPROMS Model Builder 4.1.0^®^ of a 1D model of an adiabatic packed bed reactor used for chemical looping reforming (CLR). The catalyst used for this process was 18 wt. % NiO with the support of Al_2_O_3_. A brief thermodynamic analysis using Chemical Equilibrium Application (CEA) was carried out to identify the optimum operating conditions. The model was simulated for 10 complete CL-SMR cycles. The effects of variations in temperature, pressure, gas mass velocity, nickel oxide concentration, reactor length, and particle diameter were studied to investigate the performance of the CL-SMR process under these variations. A parametric analysis was carried out for different ranges of conditions: temperatures from 600 to 1,000 K, pressure from 1 to 5 bar, gas mass velocity between 0.5 and 0.9 kg·m^−2^ s^−1^, nickel oxide concentration values between 0.1 and 1 mol·m^−3^, particle diameters between 0.7 and 1 mm, and fuel reactor (FR) lengths between 0.5 and 1.5 m. At the optimum temperature (950 K), pressure (1 bar), and steam-to-carbon molar ratio (3/1), with an increase in particle diameter from 0.7 to 1 mm, an 18% decrease in methane conversion and a 9.5% increase in hydrogen yield were observed. Similarly, with an increase in FR length from 0.5 m to 1.5 m, a delay in the temperature drop was observed.

## 1 Introduction

During the last couple of decades, global warming has emerged as one of the major problems confronting the Earth’s climate. According to an Intergovernmental Panel for Climate Change (IPCC) report, the temperature of the Earth has been rising drastically since 1850, with the last 4 decades (1980–2020) being considered the warmest (Intergovernmental panel of climate change IPCC, 2021). The main reason behind the rise in the temperature of the Earth is excessive emission of greenhouse gases (GHGs), such as N_2_O, H_2_O, CH_4_, CO_2_, SF_6_, and chlorofluorocarbons (CFCs), into the atmosphere. CO_2_ gas makes the highest contribution to GHGs, at 78% (Abbas et al., 2017). The emission of CO_2_ poses a high level of risk to the atmosphere due to its high efficiency in absorbing energy and its emission on a large scale (Rasheed et al., 2019). In 2021, 79% of total world energy was produced from fossil fuels, which emitted a total of 36.64 Gt of CO_2_, representing a 10.2% increase over CO_2_ emissions observed in 2018 (International Energy Agency IEA, 2022). Furthermore, it is expected that global energy demands will increase by up to 40% by 2040. In 2021, the total energy produced by Pakistan was 75.50 Mtoe, with CO_2_ emissions of 219.8 MT, which represents a 251.4% increase in CO_2_ emissions as compared to 1990. In comparison to 2021, it is estimated that by 2040 the consumption of natural gas for energy production will increase by up to 35% (Pakistan Energy demand forecast, 2021). With the rising energy demands, limited fossil fuel reserves, and environmental concerns, sustainable alternatives and environmentally friendly sources of energy are attracting attention and demanding greater research focus for the development of improved technology (International Energy Agency, 2017).

Hydrogen is recognized as one of the most suitable energy sources for clean energy production, as combustion of H_2_ is free of harmful pollutant emissions; due to this quality, researchers currently consider it to be the fuel of the future (Ma et al., 2016). Water vapor is the only byproduct produced along with energy production during combustion of H_2_. No harmful pollutants, such as CO_x_, SO_x_, particulate matters, or soot, are produced during combustion of H_2_ (Sharma and Ghoshal, 2015). H_2_ can be used as an energy carrier for both industrial and domestic usage. Due to its high conversion efficiency, low pollution, and recyclability, H_2_ is considered to be a perfect energy source (Liu et al., 2020). The combustion of H_2_ produces more energy per unit mass than any other fuel, including gasoline, coal, and methane (Dutta, 2014).

The processes used for production of H_2_ are gasification, pyrolysis, reforming, and electrolysis (Luo et al., 2018). At present, nearly 96% of the world’s H_2_ production is fossil fuel-based, for example, from coal, crude oil, and natural gas. Among these, natural gas is the most prominent source used for H_2_ production (Omoniyi and Dupont, 2018; Stoppacher et al., 2022). There are numerous methods used for the manufacture of H_2_ from natural gas, e.g., partial oxidation (PO_x_), steam methane reforming (SMR), and autothermal reforming (ATR). At present, approximately 75% of total H_2_ production across the world is SMR-based (Dutta, 2014). The SMR process occurs in two steps (see Appendix B) under mild pressure conditions of 20–35 atm and at an elevated temperature (between 800°C and 1,000°C). In the initial step (Appendix B, equation B1), CH_4_ is converted into H_2_; in the second step (equation B2), the water–gas shift (WGS) reaction takes place (Jin et al., 2023). The overall chemical reaction of the SMR process (Appendix B) is extremely endothermic and therefore requires an external heat source (Luo et al., 2018).

The main problem in the SMR process is the choice of oxygen transfer material (OTM), which can be tackled by maintaining appropriate specifications, such as high selectivity, high stability, and high reactivity with CH_4_, along with high resistance to carbon deposition (Pashchenko, 2018). In order from highest to lowest, the reactivity of OTMs with CH_4_ is as follows: NiO, CuO, Mn_2_O_3_, Fe_2_O_3_ (Luo et al., 2018). Metallic nickel (Ni) is most often used as a catalyst in the SMR process, and the most widely preferred OTM is also Ni-based oxide (LeValley et al., 2014). The SMR process is quite expensive, as losses in effectiveness occur with the passage of time, along with catalyst degradation (May et al., 1996).

**FIGURE 1:**
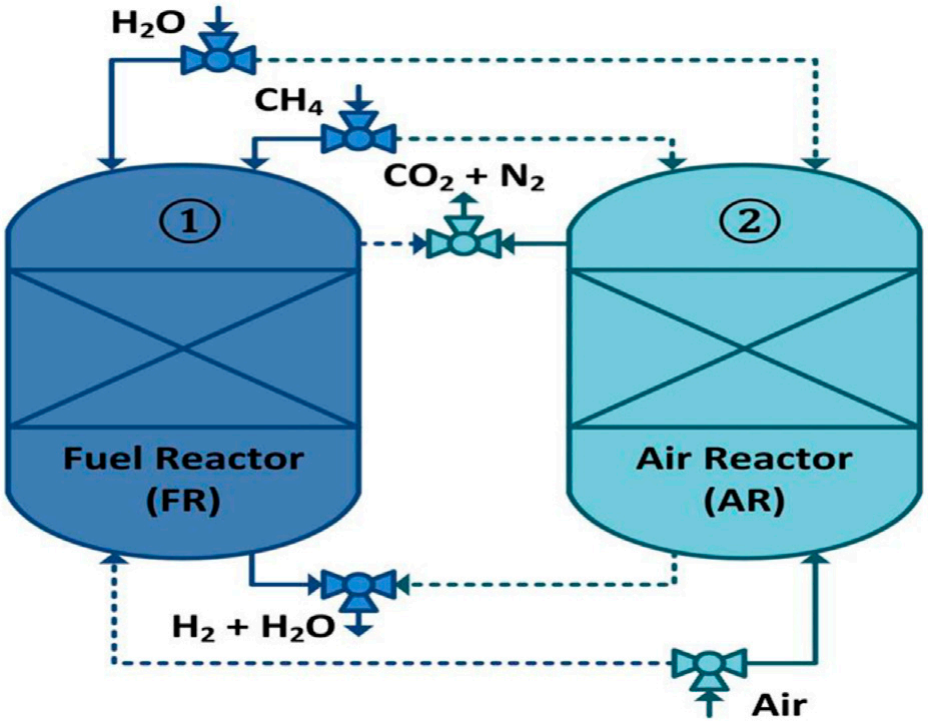
Schematic illustration of the CL-SMR process. Solid lines represent active streams, and dotted lines represent streams that are not active.

In 2000, the chemical looping reforming (CLR) technique was introduced by Lyon and Cole (Lyon and Cole, 2000). The term *chemical looping* (CL) was given to this approach due to the transportation of oxygen as part of the process. The metal reduced during SMR reactions is subsequently oxidized for the beginning of the new CL cycle; see Figure 1.

The chemical reaction equations for chemical looping–steam methane reforming (CL-SMR) are shown in Appendix B (B1 to B7 and B8 occur in a fuel reactor and an air reactor, respectively) (Luo et al., 2018). The main benefits of CL-SMR over SMR are as follows: 1) no external combustion is required; 2) steam and the catalyst are required in smaller quantities; 3) emission of sulfur pollutants is very low; and 4) there is zero formation of thermal NO_x_ (Garcia-Labiano et al., 2009; Pröll et al., 2010; Liu et al., 2020). Therefore, chemical looping technology has recently attracted increasing attention, and a great deal of research has been carried out in the development of this technology.

## 2 Literature review

In the 1950s, Lewis et al. (1951) presented the basic idea of chemical looping to produce CO_2_ and syngas by using iron- and copper-based OTMs from carbonaceous fuel. Lewis and Gilliland (Lewis et al., 1954) also introduced the idea of using two interconnected fluidized bed reactors (FBRs) for the circulation of solid particles. The concept was the same as the chemical looping combustion (CLC) process. Later, Richter et al. (1983) recommended the principle of CLC, in which they considered the metal oxides CuO and NiO as OTMs in a formation of connected FBRs to increase the efficiency of a power plant. In 1987, Ishida et al. (1987) introduced the term CLC for the first time. They reduced the exergy losses that occurred during the conversion of fuel-based energy to thermal-based energy in conventional power plants by using natural gas.

The concept of chemical looping reforming (CLR) was originally proposed in 2001 by Mattisson and Lyngfelt (2001). CLR works on the same basic principles as chemical looping combustion (CLC), but instead of thermal-based energy, the end product is H_2_. Mattisson et al. (2004) recommended that the oxygen fraction in steam should not be more than 0.3 of the total oxygen in order to maintain a high temperature and promote conversion of CH_4_.

### 2.1 Progress in oxygen carriers

Zafar et al. (2006) tested NiO, CuO, Fe_2_O_3_, and Mn_2_O_3_ on two supports, SiO_2_ and MgAl_2_O_4_, in a laboratory-scale fluidized bed reactor (FBR). They discovered that MgAl_2_O_4_ showed higher reactivity levels than SiO_2_ during redox reactions. For H_2_ production from CH_4_, Rydén et al. (2008a); Rydén (2008) worked with atmospheric and pressurized CLR processes. Based on their findings, they concluded that the pressurized process achieved 5% higher efficiency due to a reduction in the energy requirements of H_2_ compression. Additionally, they tested Fe_2_O_3_/MgAl_2_O_4_ as an OTM with addition of an NiO layer. They discovered that, with a 1% addition of NiO on the OTM surface, the reactivity increased, and thus the selectivity of CH_4_ toward H_2_ and CO. Johansson et al. (2008) studied NiO as an OTM on two different supports, MgAl_2_O_4_ and NiAl_2_O_3_. They concluded that NiO/MgAl_2_O_4_ had a lower tendency toward carbon formation and a higher tendency toward CH_4_ conversion. Diego *et al.* (Lu et al., 2018) studied the behavior of NiO as an OTM on several supports, such as γ-Al_2_O_3_, θ-Al_2_O_3_, and α-Al_2_O_3_. They determined that the OTM impregnated on α-Al_2_O_3_ showed the highest reactivity during the reduction reaction, whereas it showed the lowest reactivity on γ-Al_2_O_3_. They also observed that, with an increase in the H_2_O-to-CH_4_ ratio and a decrease in temperature, the deposition of carbon during the reduction reaction occurred. de Diego et al. (2009) also studied the CLR working process with an Ni-based OTM on α-Al_2_O_3_ and γ-Al_2_O_3_ supports in a 900 W continuous reactor. They used different operating variables, such as different solid circulation rates, H_2_O-to-CH_4_ molar ratios from 0 to 0.5, and fuel reactor (FR) temperatures between 800°C and 900°C, to analyze the effects of these variables on CH_4_ conversion and product distribution. Rydén et al. (2006); Rydén et al. (2008b); Rydén et al. (2009) tested an Ni-based OTM on different supports, such as α-Al_2_O_3_, γ-Al_2_O_3_, MgAl_2_O_4_, and ZrO_2_-MgO, in 500 W CLR continuous reactors. They achieved the complete conversion of CH_4_ and high selectivity toward H_2_ and CO in all units. Pröll et al. (2010) tested an Ni-based OTM on NiAl_2_O_4_-MgO for the CLR process in a 140 kW pilot plant. They analyzed the results in a temperature range between 750°C and 900°C. All the aforementioned studies were conducted at atmospheric pressure. Ortiz et al. (2010) studied the performance of a pressurized CLR process (up to 10 bar) in 900 W units and found results similar to those obtained with OTMs at atmospheric pressure by de Diego et al. (2009). Zainab et al. (Ibrahim et al., 2018) studied the CL reforming of shale gas using NiO on Al_2_O_3_ and CaO/Al_2_O_3_ in a packed bed reactor (PBR) at 1 bar, 750°C, and with a steam-to-carbon molar ratio of 3. They observed that significant deactivation of catalyst (NiO on CaO/Al_2_O_3_) occurred after consecutive nine redox cycles. Before catalyst deterioration, fuel conversion was above 80%, which shows that steam reforming processes are highly favored by a high temperature.

### 2.2 Progress in CLR modeling

For the purposes of scale-up, design, and optimization of the CLR process, modeling and simulation of air and fuel-based reactors would appear to be beneficial. Halabi et al. (2008) developed a 1D model of an FBR for investigation of performance in terms of conversion of CH_4_, H_2_ yield, H_2_ purity, and reforming efficiency of autothermal reforming (ATR). They assumed that the process was adiabatic in nature. Monnerat et al. (2003) developed a model for AR to determine the effect of the quantity of O_2_ on the temperature of the reactor. They also developed a model of oxidation of an Ni catalyst for unsteady-state conditions. Hoang and Chan (2004) developed a 2D model of the reformer in order to simulate the conversion behavior of the reactant. Zhou et al. (2013) developed a 1D model of a PFR for the reduction and CLC processes, using NiO as a catalyst. They assumed isothermal and isobaric conditions. Adams and Barton (2009) developed a 2D heterogeneous model of PBR for the WGS reaction. The model established could be applied to both low- and high-temperature shift reactions and was also suitable for simulation of a catalyst-based process with known kinetic data. Grigorios *et al.* (Pantoleontos et al., 2012) developed a model to examine the dynamic behavior of an industrial heterogeneous catalytic packed bed reactor (PBR) for the SMR process. The model described the physicochemical processes that take place in both the gas and solid phases, accounting for diffusional limitations within the catalyst particles.

Ghouse and Adams (2013) developed a 2D heterogeneous model of SMR. They assumed perfect mixing of the species without any carbon deposition. In their work, the equations of energy and mass transfer in the solid and gas phases were considered. Zhou et al. (2015) developed a three-phase hydrodynamic model of CL reduction with NiO as the catalyst and CH_4_ as the fuel for the analysis of experimental data. They incorporated pressure change, energy balances, mass balances, and the effect of entrainment of oxygen carriers in the freeboard region, which improves overall fuel combustion efficiency and solid conversion. They also studied the effects of mass transfer, oxygen carrier entrainment, and bubble size on the performance of the CL reducer. They found that smaller bubbles are more desirable to increase fuel combustion efficiency. Morgado et al. (2017) developed an FBR model for comparison of CH_4_ conversion, H_2_ production, the drop in temperature, and length of reactor for CLR and GSR. This model employed perfect phenomenological closures for the turbulent and fast fluidization regimes and for the bubbling phenomenon. Simulations were carried out to examine the degree of OTM consumption, which is considered to be an important process variable. According to the authors, GSR is more suitable for pure H_2_ production with integrated CO_2_ capture and CLR for power generation. Diglio et al. (2016) presented work on the numerical analysis of an ATR in a PBR with NiO as the catalyst and CH_4_ as fuel. They theoretically quantified the challenges, such as the choice of the duration of the oxidation and reduction phases, the startup temperature, and the cycle design, through numerical simulation. They concluded that suitable choices of duration for the reduction and oxidation phases and of initial temperature are essential requirements for the performance of the CLR process. Singhal et al. (2017) proposed a multiscale model of a packed bed CLR. They presented a comparison of two reactive flows at two different scales: 1) a particle-resolved direct numerical simulation, and 2) a 1D packed bed model. According to their findings, in order to utilize the model to improve an industrial-scale model, the volume of gas generated by the SMR reaction, reactant diffusion within the particles, and clear reaction order in equilibrium conditions are required.

Chenlong et al. (2019) investigated the CLR process of acetic acid by using Fe-doped LaNiO_3_ perovskites with different Ni-to-Fe ratios. They found that Ni/Fe perovskites were more stable than LaNiO_3_ perovskites, although LaNiO_3_ showed more activity in gas production. Minbeom *et al.* (Lee et al., 2020) studied the effect of transition metals at B-sites (B = Fe, Ni, Mn) of LaCoO_3_ on CL-SMR. According to their findings, Fe showed more selective oxidation of methane to syngas, the highest H_2_ purity, and the greatest extent of steam regeneration. Dragomir *et al.* (Bukur et al., 2019) investigated the redox properties of two Ni-based oxygen carriers, namely, Al and Zr. During the CLR process for successive cycles, with the help of a thermogravimetric analyzer and an *in situ* magnetometer, they found that a high degree of redox activity was seen during cyclic study of CL-SMR using the Zr-supported oxygen carrier than using the Al-supported oxygen carrier, and that the degree of redox activity increased gradually with the number of cycles. On the other hand, they observed moderate crystalline growth during Al use, while there was a decrease in crystalline size during the use of Zr.

Mathematical modeling of various sub-models, such as AR (oxidation of catalyst), FR (SMR with reduction of catalyst), and the WGS reaction of the CL-SMR process, has been reported on in the literature. However, to the best of the authors’ knowledge, model-based study of CL-SMR with NiO as a catalyst in a PBR at low pressure and with different OTM particle diameters has not yet been considered. Therefore, in this work, we considered a 1D mathematical model of the CL-SMR process in a PBR with NiO as a catalyst at low pressure (1 bar). For the implementation of the model and to determine the effects of various operating conditions, parameters such as temperature, pressure, gas mass flow velocity, void fraction, and particle diameter were considered.

## 3 Methodology

In this section, a one-dimensional heterogenous model is considered in order to understand the behavior of the physicochemical processes involved in CL-SMR; the material balance and energy balance in the gaseous and solid phases are implemented along with the model assumptions. Subsequently, a thermodynamic analysis is considered in order to determine the optimum operational conditions. Finally, the implementation of model in gPROMS, along with boundary and initial conditions, is discussed.

In the implementation of the model, the following assumptions have been adopted, in consideration of work by Abbas et al. (2017):

a) The reactor is operating under adiabatic conditions, with no heat entering or leaving the system. The main purpose of assuming the system to be adiabatic is to study the behavior of the temperature within the reactor under different conditions.
b) This model is applicable for ideal behavior, because the gases used in the system are considered to be ideal gases and equation used for these gases is the ideal gas equation.
c) Temperature change in the catalyst is not considered, as the changes within the catalyst are negligible and to consider these would makes the model very complex and sensitive. Due to this sensitivity, it would be very difficult to study the model under different operating conditions.
d) In the reactor, the plug flow pattern of gases and the temperature and concentration gradients along the length of the reactor are considered. In comparison to the axial direction, negligible changes in temperature and concentration are observed in the radial direction.

The model equations consist of equations representing material and energy balance for the gaseous phase (Eqs. 1, 2) and the solid phase (Eqs. 3, 4), and the pressure drop (Eq. 5).

$$\varepsilon_{b}\left(\frac{\partial C_{i}}{\partial t}\right)+\left(\frac{\partial uC_{i}}{\partial z}\right)+k_{g,i}\;a_{v}\;\left(C_{i}-C_{i,s}\right)=\varepsilon_{b}\;D_{z}\left(\frac{\partial^{2}C_{i}}{\partial z^{2}}\right)$$ (1)

$$\varepsilon_{b}\;\rho g\left(\frac{\partial T}{\partial t}\right)+u\rho \;C_{\text{pg}}\left(\frac{\partial T}{\partial z}\right)=h_{f}a_{v}\left(Ts-T\right)+\lambda z^{f}\;\left(\frac{\partial^{2}T}{\partial z^{2}}\right)$$ (2)

$${\text{k}}_{\text{g},\text{i}}\;{\text{a}}_{\text{v}}\;\left({\text{C}}_{\text{i}}-{\text{C}}_{\text{i},\text{s}}\right)=\left(1-\varepsilon_{\text{b}}\right)\;\rho_{\text{cat}}\;{\text{r}}_{\text{i}+}\upsilon \rho_{\text{cat}}\;{\text{r}}_{\text{i}}$$ (3)

$$\rho_{\text{bed}}\;C_{p,\text{bed}}\left(\frac{\partial T_{s}}{\partial t}\right)+h_{f}\;a_{v}\;\left(T_{s}-T\right)=\upsilon \left(1\;-\varepsilon_{b}\right)\;\rho_{\text{cat}}Ʃ-H_{\text{rxn},j}{ƞ}_{j}R_{j}$$ (4)

$$\frac{∆Pg_{c}}{L}=\left(\frac{150}{{d_{p}}^{2}}\right)\left(\frac{{\left(1-\varepsilon_{b}\right)}^{2}}{{\varepsilon_{b}}^{3}}\right)u\;\mu +\left(\frac{1.75}{d_{p}}\right)\left(\frac{{\left(1-\varepsilon_{b}\right)}^{2}}{{\varepsilon_{b}}^{3}}\right)\rho_{g}\;u^{2}$$ (5)

The supporting equations required for the calculation of the physical property terms used in mathematical model Eqs. 1–3 and Eq. 4 to Eq. 5 are provided in Supplementary Table SA; these include the dispersion coefficient (Supplementary Equation SA1), thermal conductivities (Supplementary Equations SA1, SA3), the mass transfer coefficient along with its supportive dimensionless numbers (Supplementary Equations SA1–SA7), and the heat transfer coefficient along with its supportive dimensionless number (Supplementary Equations SA8–SA11).

Additionally, the material balance for the chemical reactions (Supplementary Equations SB1–SB4) involves oxygen transfer material, as expressed by Eqs. 6, 7. It is important to mention that, in order to reduce the complexity of the model, only major chemical reactions are considered; minor or side reactions, such as methane decomposition, carbon gasification with steam, and dry methane reforming, have been neglected. With this assumption, the overall model-based results still represent the real process with error below 1% (Rasheed et al., 2019).

$$\frac{dC_{\text{Ni}}}{dt}=\left(2R_{1}+R_{2}+R_{3}+R_{4}\right)M_{\text{Ni}}$$ (6)

$$\frac{dC_{\text{Nio}}}{dt}=-\left(2R_{1}+R_{2}+R_{3}+R_{4}\right)M_{\text{NiO}}$$ (7)

In Eqs. 6, 7, *R*_1_ to *R*_4_ represent the reaction rate of the chemical reaction Supplementary Equations SB1–SB4, respectively. The required reaction rate equations for *R*_1_ (Supplementary Equation SB1), *R*_2_ (Supplementary Equation SB2), *R*_3_ (Supplementary Equation SB3), and *R*_4_ (Supplementary Equation SB4) are provided in Supplementary Appendix SB.

### 3.1 Implementation of the model

For prediction of the behavior of the reactors (fuel and air) shown in Figure 1, the differential and algebraic equations, along with boundary and initial conditions, were implemented in gPROMS Model Builder 4.1.0^®^. The initial and boundary conditions used in solving model equations 12 to 18 are provided in Table 1. The first-order BFDM was used to solve Eqs. 12–18, A.1 to A.11 (Supplementary Appendix SA), and B.1 to B.14 (Supplementary Appendix SB).

**TABLE 1:** Initial and boundary conditions required for modeling of Chemical Looping Steam methane reforming (CL-SMR).

Initial conditions	
$C_{i,o}$ /mol m^-3^ where iϵ {CH_4_, CO, H_2_, H_2_O, CO_2_ and N_2_}	[2.53, 0, 0.11, 7.6, 0, 2.53]
$C_{NiO,o}$ /mol m^-3^	0.1
$T_{o}$ /K	950
$P_{o}$ /bar	1
$T_{s,o}$ /K	950
X/%	0
Boundary conditions	
Inlet of reactor (*z* = 0)	Outlet of reactor (*z* = L)
$C_{i}=C_{i,o};T=T_{o};P=P_{o};T_{s}=T_{s,o}$	$\left(\frac{\partial C_{i}}{\partial t}\right)=0;\left(\frac{\partial T}{\partial z}\right)=0;\left(\frac{\partial T_{s}}{\partial z}\right)=0$

From Supplementary Appendix SB, it can be seen that initially (at t = 0), there is no trace of gaseous stream present in the reactor, but a small amount of H_2_ has been considered, because with a zero value the rate of reaction of the reforming reactions (equations B.5 to B.7) becomes infinite due to the denominator term in the rate equation; therefore, a minute quantity of H_2_ has been fed into the reactor along with the reactant concentration in order to simulate the reactor model thoroughly, as listed in Table 2. Additionally, the parameters required to simulate the model in gPROMS are provided in Table 2.

**TABLE 2:** Parameters used in the implementation of CL-SMR model (Abbas et al., 2017).

Parameters	Values
Void fraction, *ε*_b_/μm	0.50
Reactor length, L/m	1.50
Specific surface area per unit volume, *a*_v_/m^2^ m^−3^	300
Density of catalyst, *ρ*_cat_/kg m^−3^	550
Density of catalyst bed, *ρ*_bed_/kg m^−3^	1,625
Heat capacity of bed, Cp_bed_/J kg K^−1^	980
Viscosity of gases, μ_g_/kg m^−1^ s^−1^	0.0181E-3
Particle diameter, d_p_/m	0.0010
Avg. molecular diffusivity, D_m_/m^2^ s^−1^	1.6E-5
Gas mass velocity, G_s_/kg m^−2^ s^−1^	0.50
Thermal conductivity of gases, *λ*_g_/W m^−1^ K^−1^	3E-2
Thermal conductivity of solids, *λ*_s_/W m^−1^ K^−1^	13.80
Avg. molecular weight, M_av_/g mol^−1^	20.02
Molecular weight of Ni, M_Ni_/g mol^−1^	58.69
Molecular weight of NiO, M_NiO_/g mol^−1^	74.69
Initial specific surface area of OTM, a_o_/m^2^ kg_carrier_	102

## 4 Thermodynamic analysis of CL-SMR

To determine the optimum operational conditions of the CL-SMR process, thermodynamic analysis needs to be carried out under equilibrium conditions. In this section, the Chemical Equilibrium Application (CEA) software is used to generate the results for the equilibrium conditions.

Minimization of Gibbs free energy is the basis of the CEA software (Rydén et al., 2009). The gases species H_2_, H_2_O, CO, CO_2_, CH_4_, O_2_, N_2_, Ni, and NiO were considered in implementation of the thermodynamic analysis in CEA. The stoichiometric molar balance of N_2_ was used for calculation of the equilibrium output of each component, which further assisted in the determination of the total product moles at equilibrium. The effects of pressure and temperature on X_CH4_, H_2_ purity, and H_2_ yield were examined under equilibrium conditions with the help of CEA and the built model (Eqs. 1–7; Supplementary Appendix SA, Supplementary Equations S1–S11; Supplementary Appendix SB, Supplementary Equations B1–B14). Eqs. 8–10 were used for calculation of the fraction of $X_{{\text{CH}}_{4}}$ , H_2_ purity, and H_2_ yield, respectively.

$$X_{{\text{CH}}_{4}}=\frac{{\dot{n}}_{{\text{CH}}_{4},\text{in}}-{\dot{n}}_{{\text{CH}}_{4},\text{out}}}{{\dot{n}}_{{\text{CH}}_{4},\text{in}}}$$ (8)

$$H_{2}\text{ purity}=\frac{{\dot{n}}_{H_{2},\text{out}}}{{\dot{n}}_{{\text{CH}}_{4},\text{out}}+{\dot{n}}_{H_{2},\text{out}}+{\dot{n}}_{\text{CO},\text{out}}+{\dot{n}}_{{\text{CO}}_{2},\text{out}}}$$ (9)

$$H_{2}\text{ yield}=\frac{M_{H_{2}}\times {\dot{n}}_{H_{2},\text{out}}}{M_{{\text{CH}}_{4}}\times {\dot{n}}_{{\text{CH}}_{4},\text{in}}}$$ (10)

The performance of CL-SMR for a steam-to-carbon (S/C) molar ratio of 3 and an NiO/C ratio of 1/1 was examined to determine the effect of temperature between 600 and 1,000 K and the effect of pressure between 1 and 5 bar under equilibrium conditions with the help of CEA.

### 4.1 Outputs of chemical equilibrium of CL-SMR

**FIGURE 2:**
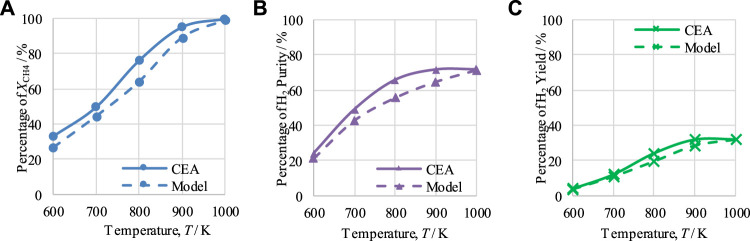
Thermodynamic analysis of the CL-SMR process at 1 bar, S/C M ratio of 3, and NiO-to-C molar ratio of 1/1, indicating the effect of temperature on **(A)** *X*_CH4_, **(B)** H_2_ purity, and **(C)** H_2_ yield.

**FIGURE 3:**
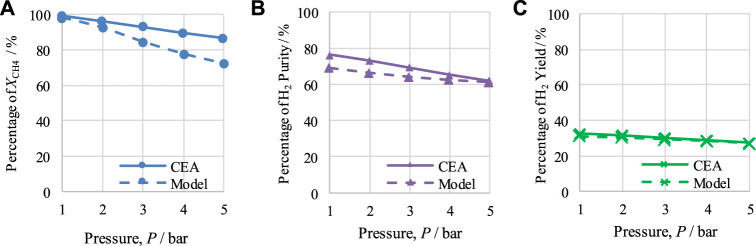
Thermodynamic analysis of the CL-SMR process at 950 K, S/C M ratio of 3, and NiO-to-C molar ratio of 1/1, indicating the effect of pressure on **(A)** *X*_CH4_, **(B)** H_2_-purity, and **(C)** H_2_-yield.

The effects of temperature and pressure on $X_{{CH}_{4}}$ , H_2_ purity, and H_2_ yield are shown in Figures 2, 3, respectively. In addition, comparisons are also made between the CEA-based and model-based results. It is important to mention that Figures 2, 3 show only the results for optimum pressure and temperature, respectively. CEA plots of CH_4_ conversion, H_2_ purity, and H_2_ yield at 600–1000 K for pressure ranging from 1 to 5 bar are presented in Supplementary Appendix SC (see Supplementary Figures C1–C3).

The results for $X_{{\text{CH}}_{4}}$ , H_2_ purity, and H_2_ yield at 1 bar are shown in Figure 2. It can be seen that, at 1 bar, increasing the temperature from 600 to 950 K also induces an increase in CH_4_ conversion, H_2_ purity, and H_2_ yield from 33% to 99.2%, 24.3%–71.5%, and 4%–32.1%, respectively. From 950 K onward, a minor change in the CH_4_ conversion value is observed, and a decline in observed for H_2_ purity and H_2_ yield. Therefore, 950 K is considered to be the optimal operating temperature for CL-SMR for attainment of maximum H_2_ purity and yield. The average difference between the CEA and model-based results was calculated by dividing the sum of the difference between CEA and model-based results by the total number of points; this resulted in differences of 5.97% for X_CH4_, 5.53% for H_2_ purity, and 1.91% for H_2_ yield.

Figure 3 shows the effects of varying pressure between 1 and 5 bar. The operating conditions for the equilibrium study of CL-SMR of 950 K, an S/C M ratio of 3, and an NiO-to-C of 1/1 are maintained. It can be seen that with the rise in the operating pressure from 1 to 5 bar the values for CH_4_ conversion, H_2_ purity, and H_2_ yield decrease from 99.2% to 86.6%, 76.5% to 61.3%, and 32.6% to 27.5%, respectively. Therefore, it can be concluded that, under the conditions implemented, the most suitable operating pressure for the CL-SMR process is 1 bar. The average difference calculated were 7.7% for X_CH4_, 4.52% for H_2_ purity, and 0.85% for H_2_ yield.

As the CEA values are based on equilibrium conditions, the model values should not be higher than those generated using CEA. Comparing the results of both studies, CEA and model-based, it can be observed from Figures 2, 3 that the model-based study values did not exceed the equilibrium values of the CEA results, falling below them in all cases; this is acceptable and proves the correctness of the model.

## 5 Results and discussion

In this section, the developed models of the FR and AR are first validated with experimental results given in the literature. Subsequently, a cyclic study of the CL-SMR process is conducted and the behavior of the gases and OTM is observed for 10 cycles. Finally, sensitivity analyses are performed for the variables C_NiO_, G_s_, and d_p_.

### 5.1 Validation of the model

This section describes the validation of the model of the CL-SMR process. Validation is performed in two steps: first, the FR model results are discussed and validated in connection with experimental results found in the literature. Subsequently, validation of the AR model is performed separately.

#### 5.1.1 Fuel reactor

**FIGURE 4:**
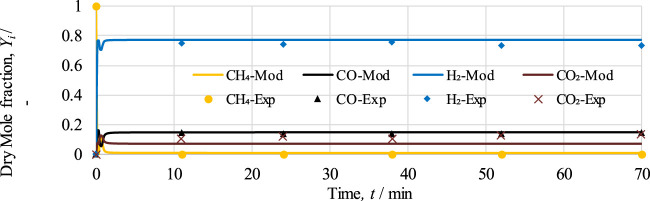
Dry mole fraction of outlet gases, $y_{i}\in \left\{{CH}_{4},H_{2},CO,{CO}_{2}\right\}$ , at 973 K, 1 bar, and S/C of 2. Solid lines represent the modeling data (MOD), while markers represent the values in the experimental data (EXP).

To authenticate the developed model of the fuel reactor (FR) considered in CL-SMR, the work of Pooya *et al.* (Azadi et al., 2011) is used. Pooya *et al.* considered 10% NiO as the OTM in an adiabatic packed bed reactor of length 60 cm and 10 cm internal diameter. The operational conditions of 973 K and 1 bar along, with 5% CH_4_ and 10% H_2_O in N_2_ feed gas, are considered for model validation. The mole fractions of gases in dry conditions obtained from experimental and model-based works are given in Figure 4. It is important to mention that during the experimental study (Azadi et al., 2011), a delay of 30 min was observed by Pooya *et al.* at beginning of the experiment. Essentially, this time delay was due to the induction period of gases in the reformer. However, for model validation, this experimental time delay has been neglected, as induction period was not considered, and values are adjusted accordingly.

In Figure 4, it can be seen that initially variation can be observed at the start of the model-based results, which is essentially due to the occurrence of reduction and SMR reactions. Once the reactions proceed, within less than 2 min, methane conversion approaches 100%, and the mole fraction of H_2_ increases from 0% to 77% and subsequently remains constant throughout the process. The average differences between model and experimental values for CH_4_, CO, H_2_, and CO_2_ are 0.9%, 0.7%, 2.7%, and 4.8%, respectively. Based on the trends, it can be determined that model-based results are in good accordance with the experimental values for the stable range of 11 min onward. However, due to the unavailability of experimental data for the time interval between 0 and 11 min, the dynamic response occurring in the experimental work cannot be compared.

#### 5.1.2 Air reactor

**FIGURE 5:**
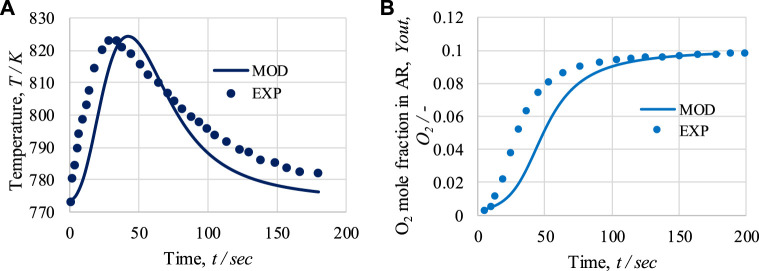
For the air reactor: **(A)** temperature profile at 1.5 bar and 10 mol% of O_2_ in feed; **(B)** O_2_ mole fraction at the outlet for 773 K, 1.5 bar, and 10 mol% of O_2_ in feed. Solid lines represent modeling (MOD) results; markers represent experimental (EXP) results.

The experimental work by Monnerat et al. (2003) used for the validation of the model in terms of OTM in the air reactor. An adiabatic packed bed reactor of length 230 mm and internal diameter 9 mm was used for oxidation of the catalyst. First, the temperature profiles from the model-based and experimental work are compared for operational pressure 1.5 bar and 10% O_2_ in feed gas; see Figure 5A. Subsequently, 10% O_2_ feed intake under temperature and pressure conditions of 773 K and 1.5 bar is considered; see Figure 5B.

As shown in Figure 5A, a rapid initial increase in temperature is observed due to the exothermic nature of the oxidation process; specifically, at the beginning of the process, all the Ni is available for oxidation. After 45 s, a decrease in temperature is observed due to the decrease in the Ni concentration. From the initial temperature of 773 K, a maximum temperature rise of 51 K is observed in the model-based work, whereas in the experimental study, a 51 K rise is observed. After 180 s of operation, the temperature reaches 776 K or 781 K in the model-based and experimental studies, respectively. The average temperature difference between the model and experimental results is 5.6 K. As shown in Figures 5A, B sudden initial rise in the amount of O_2_ at exit of the AR occurs until 70 s. After that, the slope in the curve representing the amount of O_2_ at exit of the AR decreases, becoming a horizontal straight line until 200 s. After 75 s of operation, the dry mole fraction of O_2_ at exit of the AR reaches 0.085 or 0.071 in the model-based and experimental results, respectively. The average difference in the O_2_ mole fraction between the model and experimental results is 0.012. Overall, based on the figure, it can be seen that the model results are in good agreement with the experimental work.

### 5.2 Cyclic study of the CL-SMR process

**FIGURE 6:**
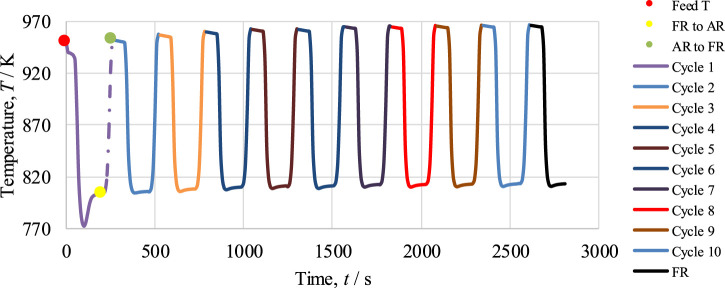
Cyclic study of the CL-SMR process for 10 cycles. The red dot indicates the start of the fuel reactor (FR) cycle; the yellow dot indicates the end of the FR cycle and start of the in reactor (AR) cycle; and the green dot indicates the end of the AR cycle.

The optimum operating conditions have already been determined by the thermodynamic analysis using CEA for the cyclic study of the CL-SMR process, as shown in Figure 1. In the FR in CL-SMR, a feed consisting of CH_4_ gas, steam, and N_2_ is introduced at 950 K, at 1 bar, and with a steam-to-carbon (S/C) molar ratio of 3/1. As shown in Figure 6, it can be observed that with entrance of the feed into the fuel reactor (FR), an immediate decrease in temperature is observed, specifically, a drop of approximately 11 K within 10 s. This decrease in temperature is due to the dominance of the endothermic nature of the reduction reaction. Subsequently, between 10 and 50 s, a further drop in temperature of 5 K is observed; in this phase, the chemical reactions, which are exothermic in nature, show their dominance along with the reduction reactions. Within less than a minute (50 s), almost 95% of the NiO is converted into Ni, catalyzing the SMR reaction. Within another minute (specifically, between 50 and 110 s), the temperature further drops; specifically, it falls by more than 10 times (from 933 to 775 K). This drop is due to the dominance of the SMR reaction, which is highly endothermic in nature. Subsequently, a rise in the temperature of the process from 775 to 803 K is observed; this rise is due to the exothermic nature of the WGS reaction. After 190 s, an overall drop in NiO % conversion and temperature can be observed; these fall by approximately 99.5% and 146 K, respectively. The yellow dot in Figure 6 indicates the end of the fuel reactor cycle, and from here onward, the air reactor cycle begins.

**FIGURE 7:**
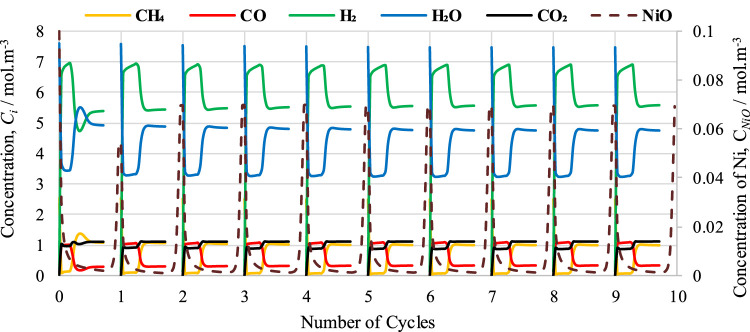
Cyclic study of outlet concentration of gases: CH_4_, CO, CO_2_, H_2_, H_2_O, and NiO.

**FIGURE 8:**
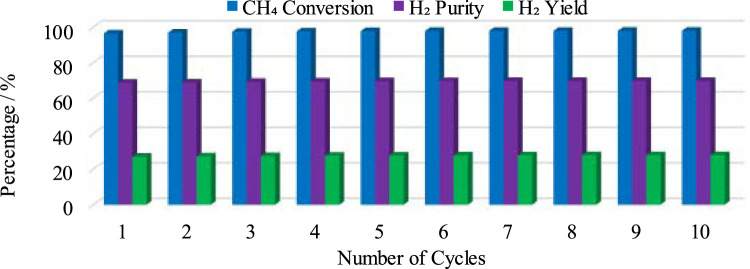
X_CH4_, H_2_ purity, and H_2_ yield during a cyclic study of the CL-SMR process for 10 cycles.

In the second part of CL-SMR, the reduced Ni is transferred into the air reactor (AR) for oxidation by injection of air (21% O_2_ and 79% N_2_) at 1 bar and 804 K. As soon as the feed has passed through the reactor, the temperature of the reactor increases from 804 to 952 K within 70 s. Subsequently, the temperature of the air reactor (AR) starts decreasing due to the decrease in the concentration of available Ni for oxidation. For reduction reaction and SMR reaction, a higher temperature is needed in the fuel reactor (FR), so the oxidation process stops when the temperature reaches 952 K. Subsequently, the feed is turned off for the AR and turned on for the FR. The green dot indicates the completion of the AR cycle, as well as the combined completion of the AR and FR cycles; see Figure 6. The FR and AR cycles together form a complete CL-SMR cycle. The CL-SMR process was studied for ten cycles. The behavior and concentrations of gases (CH_4_, CO, CO_2_, H_2_, H_2_O, and NiO) during these ten cycles are shown in Figure 7. Each cycle of CL-SMR took 280 s to complete (see Figure 6). In the initial two cycles of the CL-SMR process, these is some variation in the outlet concentrations of gases and OTM. After this point, the variation in the concentrations of gases disappears as the number of cycles increases and the process moves toward the steady state. Values for X_CH4_, H_2_ purity, and H_2_ yield for this ten-cycle study are presented in Figure 8. In every cycle, 97.5% CH_4_ conversion, 69% H_2_ purity, and an overall H_2_ yield of 27.5% were achieved.

#### 5.2.1 Comparison of outlet concentrations and reaction rates during cycles 1 and 2

**FIGURE 9:**
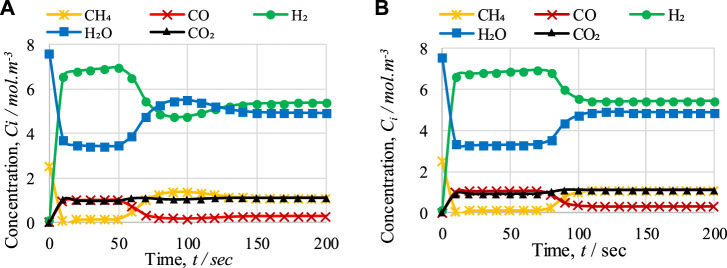
Comparison of FR outlet concentrations of gases with respect to time during **(A)** cycle 1 and **(B)** cycle 2 of CL-SMR.

The outlet concentrations of gases during cycles 1 and 2 are shown in Figure 9. In the pre-breakthrough period, both the cycles show the same kind of variation in the outlet concentrations of gases. However, during the breakthrough period (i.e., from 70 s to 110 s), both the figures show differences in the variation in the outlet concentrations of gases, mainly for H_2_ and H_2_O. After 100 s, the concentrations of H_2_ and H_2_O during cycle 1 are 4.77 mol·m^−3^ and 5.49 mol·m^−3^, respectively, while during cycle 2, the concentrations of H_2_ and H_2_O are 5.56 mol·m^−3^ and 4.73 mol·m^−3^, respectively; this inconsistency is due to the difference in temperature drop between the cycles. In Figure 6, it can be seen that after 100 s of operation, the temperature drops to 772 K in cycle 1 and 804 K in cycle 2. This difference of 32 K is the main reason for the difference in the variation in the outlet concentration of gases, because the optimum temperature range for the SMR reaction is 900–1,100 K. As shown in Figures 9A, B, it can be observed that in the pre-breakthrough period, a drop of 2.44 mol·m^−3^ and 3.9 mol·m^−3^ (cycle 1) and 2.442 mol·m^−3^ and 4.3 mol·m^−3^ (cycle 2) occurs in the concentration of CH_4_ and H_2_O, respectively. In contrast, a rise of 6.59, 0.97, and 0.98 mol·m^−3^ (cycle 1) and 6.61, 1.03, and 0.92 mol·m^−3^ (cycle 2) can be observed in the concentration of H_2_, CO, and CO_2_, respectively.

**FIGURE 10:**
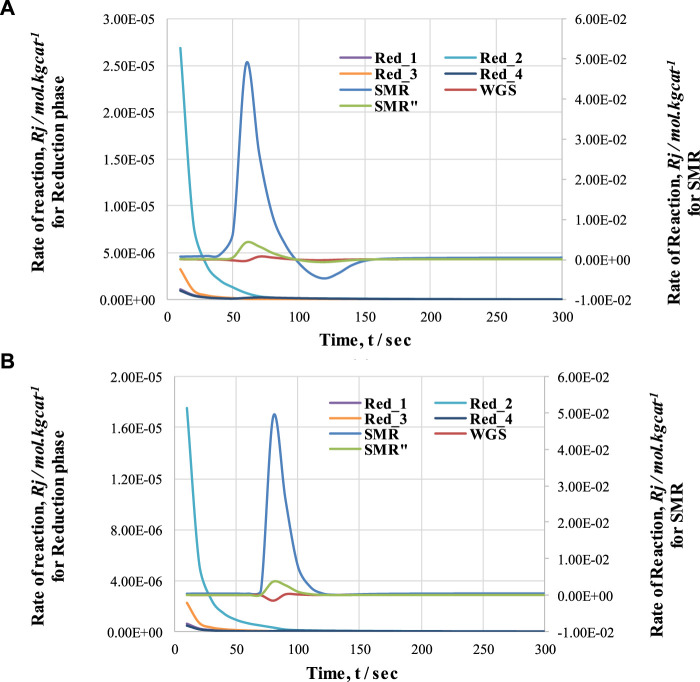
Comparison of rate of reactions (SMR and reduction) with respect to time during **(A)** cycle 1 and **(B)** cycle 2 of CL-SMR.

Figure 10 shows the rate of reactions for cycle 1 and cycle 2. This graph indicates that the reduction reactions are so fast that they show variation in the reaction rate within the first 20 s of the process, and convert all the NiO into Ni. The drop and rise in the concentrations of gases during first the 20 s is due to the activation of reduction reactions; in these reactions, NiO reacts with CH_4_ to form H_2_, CO, and CO_2_. A decrease in CH_4_ concentration and increases in H_2_, CO, and CO_2_ concentration can be observed. Subsequently, until 60 s, the change in gas concentrations remains constant; at this point, the pre-breakthrough period ends and the breakthrough period begins. Unlike the pre-breakthrough period, during the breakthrough period the concentrations of CH_4_ and H_2_O increase, whereas the concentration of H_2_ decreases. This large change in concentration is due to the activation of the SMR reaction, which is highly endothermic. The SMR reaction is more dominant than the other two reactions (WGS and overall SMR) here. This change in reaction rate also indicates the reason for the change in concentrations of outlet gases from the FR.

### 5.3 Sensitivity analysis

In this section, a sensitivity analysis is carried out by observing the impact of concentration of NiO (C_NiO_) and gas mass velocity (G_s_) on the temperature profile of the CL-SMR process, as well as the impact of particle diameter (d_P_) and reactor length L on CH_4_ conversion, H_2_ purity, and H_2_ yield.

#### 5.3.1 Effect of C_NiO_ and G_s_

**FIGURE 11:**
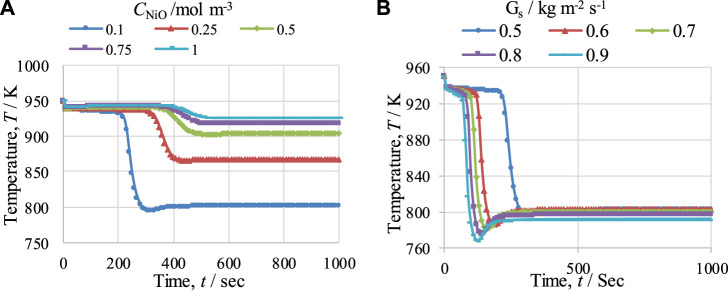
Temperature profiles for the fuel reactor (FR) at 950 K, at 1 bar, and with a steam-to-carbon molar ratio of 3, showing the effects of **(A)** NiO concentration (C_NiO_) and **(B)** gas mass flow velocity (G_s_).

The effects of NiO concentration (*C*_NiO_) and *G*_*s*_ on the temperature of the fuel reactor (FR) were studied; the results are presented in Figures 11A, B, respectively. In Figure 11A, it can be seen that with a rise in the NiO concentration, the duration of the pre-breakthrough period increases because of the greater amount of NiO available for reduction reactions, but the drop in temperature decreases, e.g., from 155 to 24 K for a change in NiO concentration in the FR from 0.1 to 1 mol·m^−3^. The reduction in the temperature drop is due to the smaller amount of CH_4_ available for the SMR reaction, which is highly endothermic in nature, as more CH_4_ is consumed during the reduction reactions because of the larger amount of NiO. In contrast, in Figure 11B, it can be seen that with an increase in the value of *G*_*s*_ from 0.5 to 0.9 kg m^−2^ s^−1^, the duration of the pre-breakthrough period decreases, as the reactants remain lower in the reactor at the higher value of *G*_*s*_. With an increase in *G*_*s*_, the drop in temperature increases from 150 to 190 K. This is because the availability of gas for the SMR reaction per unit area and time is enhanced with the increase in *G*_*s*_, and so the temperature drop also increases. Similarly, at lower values of Gs, more CH_4_ is consumed before the breakthrough period in the reduction reaction and less CH_4_ is available for the SMR reaction, which reduces the temperature drop during the breakthrough period.

#### 5.3.2 Effect of particle diameter

**FIGURE 12:**
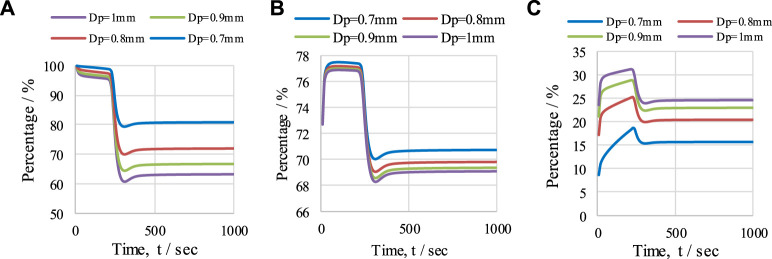
The effect of OTM particle diameter (0.7–1 mm) under operating conditions of 950 K, with S/C M ratio of 3, on **(A)** *X*_*CH4*_, **(B)** H_2_ purity, and **(C)** H_2_ yield. Effect of reactor length (L) on temperature (T) profile.

The effect of OTM particle diameter was studied to observe the overall performance of the CL-SMR process. The operating conditions of 950 K, 1 bar, and an S/C M ratio of 3 were maintained. The values of X_CH4_, H_2_ purity, and H_2_-yield, for particle diameters ranging from 0.7 to 1 mm, are presented in Figure 12. A decrease in X_CH4_ from 81% to 63% is observed as the diameter of the particles increases from 0.7 to 1 mm; see Figure 12A. Similarly, a minor decrease in H_2_ purity (see Figure 12B) from 71% to 69% is also observed with this increase in the OTM particle diameter. This is because, with an increase in the size of OTM particles, less surface area is available to the gases for reaction, and therefore reductions in CH_4_ conversion and H_2_ purity is detected. The size of the OTM particle is inversely proportional to CH_4_ conversion and H_2_ purity. On the other hand, H_2_ yield in the CL-SMR process increases from 15.5% to 25% (see Figure 12C) with the increase in particle diameter from 0.7 to 1 mm. This increase in H_2_ yield is due to more formation of H_2_ per unit mole of CH_4_ entering the reactor. However, in the reactor, the variation in X_CH4_, H_2_ purity, and H_2_-yield over time directly relates to the temperature variation.

#### 5.3.3 Effect of Reactor Length (L) on Temperature (T) profile

**FIGURE 13:**
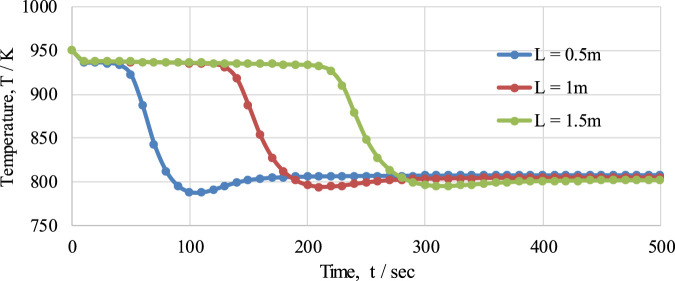
The effect of different fuel reactor lengths (0.5 m, 1 m, and 1.5 m) on the temperature profile of the fuel reactor in the CL-SMR process.

The effect of FR length on temperature variation in the reactor at pressure of 1 bar, a temperature of 950K, and with an S/C ratio of 3 over a given period of time is presented in Figure 13. The behavior of the temperature profile in the reactor was examined for three different lengths of reactor (namely, 0.5 m, 1 m, and 1.5 m). From the graph, it can be seen that with an increase in the length of the FR, a delay in the temperature drop is observed, while the temperature profiles were almost identical for all lengths of reactor. This delay in temperature drop is because of the time taken to consume the oxygen carrier during the reduction reaction in the reactor: as the length of the reactor increases, the length of the bed of OTM also increases. The maximum temperature drops for 0.5 m, 1 m, and 1.5 m length are 163 K, 157 K, and 152 K, respectively; similarly, the amounts of time taken to reach the steady state are 200 s, 300 s, and 400 s, respectively, as shown in the figure.

## 6 Conclusion and outlook

A one-dimensional simulation of a heterogenous catalytic CL-SMR process in an adiabatic PBR, at low pressure, was conducted in gPROMS Model Builder^®^. First, a thermodynamic analysis of the process was carried out, using CEA, to identify the optimum temperature (950 K) and pressure (1 bar) conditions for the CL-SMR process at an S/C M ratio of 3. Next, the effects of temperature and pressure on X_CH4_, H_2_ yield, and H_2_ purity in the CL-SMR process were studied at equilibrium conditions; the findings were compared with the results of the validated model. The effects of increasing temperature (from 600 to 1000 K) and pressure (from 1 to 5 bar) on X_CH4_, H_2_ yield, and H_2_ purity in the CL-SMR process were positive and negative, respectively. This adiabatic model of the CL-SMR process was run for 10 cycles. It was observed that during each cycle the changes in the values of X_CH4_, H_2_ purity, and H_2_ yield were negligible.

The effect of reaction rate, along with a comparison of the first two cycles of CL-SMR, were also presented. The behavior of temperature in the FR was examined for different values of Gs (0.5–0.9 kg m−^2^ s−^1^) and C_NiO_ (0.1–1 mol m−^3^). It was concluded that, with an increase in the value of G_s_, the delay in the temperature drop or the duration of pre-breakthrough period was decreased. An increase in the concentration of NiO was found to reduce the temperature drop in the FR. The effect of OTM particle diameter on CL-SMR performance was also studied. It was concluded that, with decrease in particle size from 1 to 0.7 mm, there was an increase in X_CH4_ and H_2_ purity, but a decrease in H_2_ yield. Finally, the effect of reactor length on the temperature variation profile within the fuel reactor was also studied, with three different lengths examined (0.5 m, 1 m, and 1.5 m). With an increase in the length of the FR, a delay in the temperature drop and activation of the SMR was observed, while the behavior of the temperature profile remained the same for each length. From this study, it can be concluded that the model developed here is effective and that the process runs at optimum temperature and pressure values for 10 cycles without any change in OTM concentration or products.

## Figures and Tables

**Table:** 

***a***	Specific surface area/m^2^ kg_carrier_^−1^
***C***	Concentration/mol m^−3^
***C***_***p***_	Heat capacity/J kg^−1^ K^−1^
***D***	Dispersion coefficient/m^2^ s^−1^
***d***	Diameter/mm
**D**_**m**_	Molecular Diffusivity/m^2^ s^−1^
***E***	Activation energy/J mol^−1^
*G*	Gibbs free energy/J
***Gs***	Gas mass velocity/Kg m^−2^ s^−1^
***H***	Heat of reaction/J mol^−1^
**Δ*H***	Heat of adsorption/J mol^−1^
***hf***	Gas to solid heat transfer coefficient/W m^−2^ s^−1^
**jD**	Chilton–Colburn factor of heat transfer
***jH***	Chilton–Colburn factor of mass transfer
***K***	Adsorption constant
***k***	Kinetic rate constant
***K***_***g***_	Gas to solid mass transfer coefficient/m^3^ m^−2^ s^−1^
***Kj***	Thermodynamic equilibrium constant
***k***_***o***_	References temperature dependent kinetic rate constant
***P***	Total gas pressure/bar
***Pr***	Prandtl number
***R***	Rate of reaction/mol kg_cat_^−1^ s^−1^
***r***	Rate of formation or consumption/mol kg_cat_^−1^ s^−1^
***Re***	Reynolds number
***R***_***g***_	Ideal gas constant/J mol^−1^ K^−1^
***S***_***ci***_	Schmitt number
***T***	Temperature/K
***u***	Velocity of gases/m s^−1^
***X***	Conversion/- Molecular diffusivity/m^2^ s^−1^
***Greek letters***
***ƞ***	Effectiveness factor
***ρ***	Density/kg m^−3^
***ε***	Porosity
**Ω**	Unitless term in reaction kinetics
***µ***	Gas viscosity/kg m^−1^ s^−1^
***λ***	Thermal conductivity/W m^−1^ k^−1^
***Subscripts***
**b**	Bed
**cat**	Catalyst
**g**	Gas phase
***i***	Gas species
***j***	No. of reaction
**o**	Initial value
***p***	particle
**rxn**	Reaction
**s**	Solid phase
**z**	Axial direction

## Data Availability

The original contributions presented in the study are included in the article/Supplementary Material, further inquiries can be directed to the corresponding author.
